# Diagnosis of Fatal Human Case of St. Louis Encephalitis Virus Infection by Metagenomic Sequencing, California, 2016

**DOI:** 10.3201/eid2310.161986

**Published:** 2017-10

**Authors:** Charles Y. Chiu, Lark L. Coffey, Jamie Murkey, Kelly Symmes, Hannah A. Sample, Michael R. Wilson, Samia N. Naccache, Shaun Arevalo, Sneha Somasekar, Scot Federman, Doug Stryke, Paul Vespa, Gary Schiller, Sharon Messenger, Romney Humphries, Steve Miller, Jeffrey D. Klausner

**Affiliations:** University of California, San Francisco, California, USA (C.Y. Chiu, H.A. Sample, M.R. Wilson, S. Arevalo, S. Somasekar, S. Miller);; University of California, San Francisco–Abbott Viral Diagnostics and Discovery Center, San Francisco (C.Y. Chiu, S. Arevalo, S. Somasekar, S. Miller);; University of California, Davis, California, USA (L.L. Coffey, K. Symmes);; University of California, Los Angeles, California, USA (J. Murkey, S. Federman, D. Stryke, P. Vespa, G. Schiller, R. Humphries, J.D. Klausner);; University of Southern California, Los Angeles (S.N. Naccache);; California Department of Public Health, Richmond, California, USA (S. Messenger)

**Keywords:** St. Louis encephalitis virus, SLEV, West Nile virus, Zika virus, flavivirus infections, arboviruses, mosquitoborne infections, vector-borne infections, outbreak surveillance, metagenomic next-generation sequencing, mNGS, whole-genome viral sequencing, viruses, California, United States, meningitis/encephalitis

## Abstract

We used unbiased metagenomic next-generation sequencing to diagnose a fatal case of meningoencephalitis caused by St. Louis encephalitis virus in a patient from California in September 2016. This case is associated with the recent 2015–2016 reemergence of this virus in the southwestern United States.

St. Louis encephalitis virus (SLEV), in the *Flaviviridae* family, is an infectious RNA virus transmitted by *Culex* spp. mosquitoes (*1*,*2*). Clinical manifestations range from mild febrile illness to fatal neurologic disease. According to recent reports (*3*,*4*), SLEV reemerged in the summer of 2015 in California and Arizona, USA, after a documented 11-year absence of activity in California.

In June 2016, we launched a multi-institutional clinical study titled Precision Diagnosis of Acute Infectious Diseases (PDAID). This 1-year study aimed to enroll 300 patients to evaluate the clinical utility of a metagenomic next-generation sequencing (mNGS) assay for diagnosing infectious causes of meningitis and encephalitis from patient cerebrospinal fluid (CSF) samples (*5*,*6*). The mNGS assay uses an unbiased sequencing approach to comprehensively identify pathogens (viruses, bacteria, fungi, and parasites) in clinical samples (*7*). We report a fatal human case of SLEV infection diagnosed by mNGS in a PDAID study patient from California.

## The Case

The case-patient was a 68-year-old man with a history of coronary artery disease, hypertension, and mantle cell lymphoma treated with 4 cycles of chemotherapy and granulocyte colony stimulating factor. He sought medical care at the end of August 2016 having had 2 days of fever (up to 39.4°C), chills, lethargy, and confusion. He had fallen twice because of dizziness and reported shortness of breath, cough, and new-onset urinary incontinence.

The patient was a retired oil-field worker living with his wife in Bakersfield, Kern County, California. He owned 1 dog and had frequent contact with his 10 grandchildren. His travel history was notable for a trip to “the mountains” in late April 2016 (Payson, Arizona, elevation 1,500 m).

The patient was admitted to the hospital in early September 2016. An initial workup, including magnetic resonance imaging of the brain, was unrevealing. Empirical therapy with vancomycin, meropenem, and levofloxacin was started after lung imaging revealed inflammatory pneumonitis. On hospitalization day 3, the patient became acutely hypoxic with worsening altered mental status (AMS), and he was intubated and transferred to the intensive care unit. A lumbar puncture revealed CSF pleocytosis (18 leukocytes/mm^3^; 35% monocytes, 33% lymphocytes, and 32% neutrophils); glucose and protein were within reference ranges. Empiric antibiotic therapy was continued, and acyclovir and antifungal therapy were added to his regimen. Repeat lumbar puncture performed on hospital day 9 showed persistent pleocytosis. All microbiologic test results for CSF, blood, and bronchoalveolar lavage were negative (Table 1), as was a workup for noninfectious causes (Technical Appendix).

**Table 1:** Microbiologic testing results for a patient with fatal mosquito-borne St. Louis encephalitis virus infection diagnosed by metagenomic sequencing, California, 2016*

Test	Hospitalization day sample collected	Result
Serum studies		
Bacterial cultures	0, 2, 4, 7	Negative
Fungal cultures	0, 2, 4, 7	Negative
Mycobacterial culture	10	Negative
*Aspergillus* antigen EIA	5	Negative
Adenovirus PCR	19	Negative
CMV DNA quantitative PCR	4, 12	Negative
EBV DNA quantitative PCR	10	Negative
Enterovirus RNA	19	Negative
HSV-1 and HSV-2 PCR	12	Negative
HHV-6 PCR	19	Negative
HIV RNA quantitative PCR	5	Negative
HBV DNA quantitative PCR	9	Negative
*Leptospira* DNA	14	Negative
Parvovirus B19 DNA	19	Negative
VZV DNA, qualitative PCR	19	Negative
Cryptococcal antigen	5	Negative
CSF studies		
HSV 1 and 2 PCR	3	Negative
Fungal culture	3	Negative
Bacterial culture	3	
*Coccidioides* Ab CF, ID	8	Negative
CMV PCR	9	
EBV PCR	3, 9	Negative
HHV-6 PCR	3	Negative
JC polyomavirus DNA, PCR	9	Negative
Mycobacterial culture	9	Negative
*Mycobacterium tuberculosis* DNA PCR	10	Negative
Meningoencephalitis antibody panel†	10	Negative
VDRL	9	Negative
VZV Ab IgG	9	Negative
West Nile virus RNA	9	Negative
mNGS for pathogen detection	9	SLEV
Respiratory secretion testing‡		
Bacterial culture	4, 5, 8	*Candida albicans*
Fungal culture	5, 8	*C. albicans*
Respiratory virus panel§	4 (NP swab), 5	Negative
*Mycoplasma pneumoniae* PCR	5	
HSV-1 and HSV-2 PCR	5	Negative
CMV PCR	5	Negative
*Pneumocystis* DFA	5	Negative
Mycobacterial culture	5	Negative
*Legionella* culture and urinary Ag	5	Negative
*Nocardia* culture	8	Negative
Serologic testing		
*Coccidioides* IgG/IgM	4, 9	Negative
*Coccidioides* complement fixation	8	Negative
HCV Ab	9	Negative
HBV, core Ab and hepatitis B e Ab	9	Negative
*Mycobacterium tuberculosis* quantiferon gold	8	Negative
Q fever antibody	8	Negative
Rapid plasma reagin	10	Negative


*Ab, antibody; Ag, antigen; BAL, bronchoalveolar lavage; CF, complement fixation; CMV, cytomegalovirus; DFA, direct fluorescent antigen; DNA, deoxyribonucleic acid; EBV, Epstein-Barr virus; EIA, enzyme immunoassay; HBV, hepatitis B virus; HCV, hepatitis C virus; HHV-6, human herpesvirus 6; HIV, human immunodeficiency virus; HSV, herpes simplex virus; ID, immunodiffusion; IFA, indirect fluroescent antibody; mNGS, metagenomic next-generation sequencing; NP, nasopharyngeal; RNA, ribonucleic acid; SLEV, St. Louis encephalitis virus; VRDL, Venereal Disease Research Laboratory; VZV, varicella zoster virus. †Includes IgM and IgG testing for West Nile virus; California encephalitis virus; Eastern equine encephalitis virus; St. Louis encephalitis virus; Western equine encephalitis virus; lymphocytic choriomeningitis virus; herpes simplex virus types 1 and 2 (HSV-1 and 2); adenovirus; influenza A; influenza B; measles (IFA); mumps (IFA); varicella-zoster Ab CF; coxsackie A types 2, 4, 7, 9, 10, and 16; coxsackie B types 1, 2, 3, 4, 5, and 6; echovirus types 4, 7, 9, 11, and 30; and CMV.  ‡Testing performed on bronchoalveolar lavage unless noted otherwise. §Detects the following viruses: influenza A; influenza A H1 seasonal; influenza A H3 seasonal; influenza A 2009 H1N1; influenza B; respiratory syncytial virus A and B; parainfluenza viruses 1–4; human metapneumovirus; human rhinovirus; adenovirus serogroups C and B/E; coronaviruses NL63, HKU1, 229E, and OC43.

After enrolling the patient in the PDAID study, we analyzed CSF from hospitalization day 9 by mNGS testing at University of California, San Francisco (Technical Appendix) (*8*). RNA and DNA sequencing libraries from CSF yielded 8,056,471 and 9,083,963 sequence reads, respectively. In the RNA library, 236,615 (2.9%) of the reads were identified as SLEV by using the SURPI+ (sequence-based ultra-rapid pathogen identification) computational pipeline (*7*), with recovery of 99.4% of the predicted 10,936-bp virus genome. Subsequent mNGS testing of the patient’s CSF from hospitalization day 3 also was positive for SLEV.

The patient’s SLEV genome sequence was >99% identical with previously sequenced 2014–2015 SLEV isolates from mosquitoes in California and Arizona (*4*). Phylogenetic analysis placed the patient’s strain in a cluster containing these isolates and viruses sequenced from mosquitoes in Argentina in 1978 and 2005 (*9*) (Figure). The patient’s SLEV was genetically distinct from the 2003 Imperial Valley strain that had been circulating in California before an 11-year absence (*12*), suggesting that he was infected by the 2015–2016 reemergent genotype currently circulating in the southwestern United States (*3*,*4*). Furthermore, the patient’s SLEV genome was closely related to a strain sequenced from a mosquito pool collected in June 2016 from Kern County (Figure, panel A), with 99.9% pairwise nucleotide identity and only 5 single-nucleotide variants across the genome.

**Figure:**
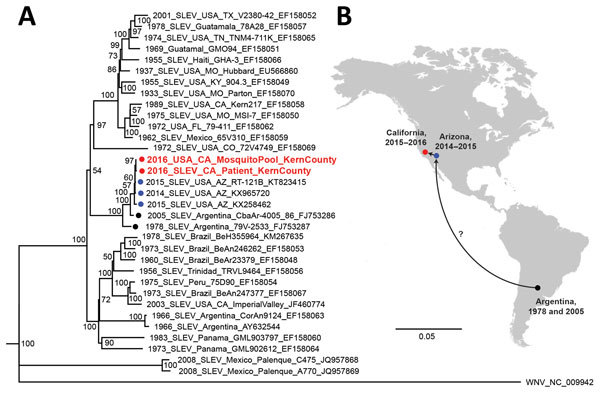
Phylogeny and spread of St. Louis encephalitis virus. A) Multiple sequence alignment of 32 complete SLEV genomes from GenBank and the 2 SLEV genomes corresponding to the case-patient’s strain and a strain from a mosquito collected in June 2016 from Kern County, California (red circles and text). Alignment was performed using MAFFT (*10*), followed by tree generation using a neighbor-joining algorithm using Geneious (*11*). The cluster containing the 2014–2016 California and Arizona SLEV genome, including those from the case-patient and 2016 mosquito pool, is rooted by SLEV strains sequenced from mosquitoes collected in Argentina in 1978 and 2005 (black circles). Isolates are named by location, year of collection, strain name, and GenBank accession number. Bootstrap support values are given for each node. Scale bar indicates nucleotide substitutions per site. B) Geographic spread of SLEV in the Americas, from Argentina in 2005 to California and Arizona during 2014–2016. Because genome sequences from US states reporting SLEV activity are not publicly available and surveillance for SLEV in South and Central America is not routinely performed, the pathway or pathways by which the virus came to the southwestern United States remain unclear (question mark). SLEV, St. Louis encephalitis virus.

After extensive discussion with his wife regarding the patient’s SLEV diagnosis and poor prognosis, the patient was transitioned to comfort care on hospitalization day 23 and died the following day. Autopsy revealed residual mantle cell lymphoma and bronchopneumonia consistent with infection or chemical pneumonitis from aspiration. The diagnosis of SLEV meningoencephalitis was subsequently confirmed by positive reverse transcription PCR and virus culture testing from multiple laboratories (Table 2). However, follow-up testing for SLEV from the patient’s CSF and serum was negative.

**Table 2:** Results of follow-up confirmatory testing for SLEV after mNGS result for a patient with fatal mosquito-borne St. Louis encephalitis virus infection diagnosed by metagenomic sequencing, California, 2016*

Test (hospital day sample collected)	Laboratory	Result
CSF studies		
SLEV, RT-PCR (9)	UCSF research lab	Positive
SLEV, RT-PCR (3,9)	CDPH	Positive
SLEV, RT-PCR (3,9)	CDC	Positive
Viral culture (3,9)	CDC	Positive, confirmed as SLEV by RT-PCR
SLEV, IgG/IgM antibody (3,9)	Quest Diagnostics	Negative, <1:10
SLEV, PRNT for neutralizing antibodies (9)	CDPH	Negative, <1:10
WNV, IgM		
WNV, PRNT for neutralizing antibodies (9)	CDPH	Negative, <1:10
Serum studies		
SLEV, RT-PCR (23)	CDPH	Negative
SLEV, IgM antibody (23)	CDPH	Negative, <1:10
WNV, IgM antibody (23)	CDPH	Negative, <1:10
SLEV, PRNT for neutralizing antibodies (23)	CDPH	Negative, <1:10
WNV, PRNT for neutralizing antibodies (23)	CDPH	Borderline positive, 1:10 (normal <1:10)

*Tests were performed after mNGS testing of patient CSF was positive from aliquots collected on hospital days 3 and 9. CDC, Centers for Disease Control and Prevention; CDPH, California Department of Public Health; CSF, cerebrospinal fluid; mNGS, metagenomic next-generation sequencing; PRNT, plaque-reduction neutralization testing; RT-PCR, reverse transcription PCR; SLEV, St. Louis encephalitis virus; UCSF, University of California, San Francisco; WNV, West Nile virus.

## Conclusions

We present a case of SLEV infection in an elderly immunocompromised patient hospitalized with fever and AMS and who experienced critical respiratory failure. Most SLEV infections are asymptomatic; when infections are symptomatic, clinical features include fever, lethargy, and confusion (*1*), with potential complications including sepsis, gastrointestinal hemorrhage, pulmonary embolism, and bronchopneumonia. In hindsight, SLEV infection is consistent with the patient’s clinical presentation, with pneumonitis and respiratory decompensation possibly resulting from aspiration during the patient’s AMS from viral meningoencephalitis. Deaths from SLEV infection during the first 2 weeks are generally from encephalitis, whereas later deaths are more often caused by complications of hospitalization, such as this patient’s bronchopneumonia.

Routine diagnosis of SLEV is challenging because serologic testing is only performed by specialized reference laboratories, the period of viremia is brief, and molecular testing by reverse transcription PCR is not widely used. Clinicians in California might fail to consider SLEV when examining a patient with nonspecific febrile illness, especially given the lack of virus or disease activity in the state during 2004–2015. Antibody testing can be complicated by the absence of seroreactivity in elderly and immunocompromised patients, as observed in the case of this patient (Table 1), as well as potential cross-reactivity with other flavivirus infections, such as dengue, Zika virus, and West Nile virus (*3*).

The identification of SLEV infection in CSF by using a panpathogen metagenomic sequencing assay is another demonstration of the power of an unbiased approach to infectious disease testing (*5*–*7*), although challenges remain with respect to test availability, interpretation, and validation (*8*). No antiviral therapy for SLEV has been proven to be efficacious, although interferon-α has been tried (*13*). With a laboratory sample-to-reporting time of 4 days, earlier sample submission might have spared our patient from the side effects of antimicrobial drug therapy, costly laboratory testing, and invasive procedures. Importantly, the family obtained reassurance and closure from communication of an established diagnosis.

During summer 2016, SLEV was reported in mosquitoes from 7 counties in California, including Kern County, where the patient resided (*4*). According to his wife, the patient often sat outdoors during the few weeks before hospitalization, although she did not recall his reporting any mosquito bites. Nevertheless, we believe he most likely contracted SLEV in California, because his history of travel to Arizona 5 months prior was not consistent with the incubation period of the disease (4–21 days); mosquitoes are less prevalent at the higher altitudes of Payson, Arizona; and the patient’s SLEV sequence was most closely related to a strain from a June 2016 Kern County mosquito pool. Given the reemergence of SLEV in the southwestern United States, clinicians from affected areas will need to maintain a high index of suspicion for this disease, particularly during local community outbreaks or high SLEV activity detected through mosquito surveillance efforts.

Technical Appendix Additional description of diagnostic workup and methods used in study of fatal human case of St. Louis encephalitis virus infection.
