# Supplementary material for: Diagnosis of Fatal Human Case of St. Louis Encephalitis Virus Infection by Metagenomic Sequencing, California, 2016
Source: Emerg Infect Dis. 2017 Oct;23(10):1964–8. doi: 10.3201/eid2310.161986 (PMC5621550; doi:10.3201/eid2310.161986)
Supplement: Technical Appendix — Additional description of diagnostic workup and methods used in study of fatal human case of St. Louis encephalitis virus infection. [file 16-1986-Techapp-s1.pdf]

Article DOI: https://doi.org/10.3201/eid2310.161986

Diagnosis of Fatal Human Case of St. Louis Encephalitis Virus Infection by Metagenomic Sequencing, California, 2016

Technical Appendix

Diagnostic Workup for Noninfectious Etiologies of Patient's Illness

Prior to admission, the patient was noted to have hyperleukocytosis in an outpatient clinic with a peripheral leukocyte (leukocyte) count of 92/mm^3. This was attributed to his cancer treatment, and he underwent urgent leukopheresis with mild symptomatic improvement.

On hospital admission, toxicity from his chemotherapeutic regimen, in particular Ara-C, was raised as a potential etiology for the patient's altered mental status (AMS), but was considered unlikely since he had not been administered this drug in the past 2 weeks and had not had any prior episodes of AMS in association with this drug. A transthoracic echocardiogram obtained due to dyspnea showed a mildly reduced cardiac ejection fraction of 45%–50%, and the patient was treated with diuretics. Head computed tomography scanning for an acute stroke was negative. Given his acute hypoxemia and risk of pulmonary embolism in the setting of malignancy, he was empirically started on anticoagulation, which was discontinued after a high-resolution computed tomography scan of the chest was negative.

Given a high serum ferritin level (>13,000), acute transaminitis, hypertriglyceridemia, and persistent AMS, the possibility of hemophagocytic lymphohistiocytosis (HLH) was raised. A bone marrow biopsy was performed on hospital day 11, but pathology findings were unremarkable, as were studies for heavy metal exposures (e.g., iron and copper). Although autoimmune testing by a paraneoplastic panel was negative, the patient was given 2 days of empiric intravenous immunoglobulin (IVIg) starting on hospital day 16, with mild improvement in mental status. However, the IVIg was discontinued the following day when he acutely decompensated with hypoxemia, hypertension, and flash pulmonary edema. He was treated with aggressive diuresis and antihypertensive medications and re-institution of IVIg with no

improvement. A repeat EEG was abnormal with possible seizure activity, so the patient was started on levetiracetam, with improvement in the EEG but no improvement in his mental status.

Sample Collection and Ethics

After obtaining informed consent from the patient's family under protocols approved by the institutional review boards of UCLA and UCSF, the patient was enrolled in the PDAID study. CSF samples from hospital days 3 and 9 were submitted to UCSF for mNGS testing. The initially tested sample was processed from hospital days 20 to 23, and the results communicated to the providers and patient's family on hospital day 23.

Metagenomic Library Construction

Separate DNA and RNA metagenomic libraries were constructed and sequenced from the patient's CSF sample according to a standardized operating procedure (SOP) in a Clinical Laboratory Improvement Amendments (CLIA)-certified microbiology laboratory as previously described (*1*). For each sequencing run, the SOP includes running two external controls (*1*): a negative "no-template" control (NTC) sample consisting of elution buffer (*2*), and a positive control (PC) sample consisting of a mixture of 7 representative pathogens (CMV, HIV, *Streptococcus agalactiae*, *Klebsiella pneumoniae*, *Cryptococcus neoformans*, *Aspergillus niger*, and *Toxoplasma gondii*) spiked at concentrations 1–2 log above the estimated limits of detection. Each sample also contains two internal spiked bacteriophage controls for RNA and DNA corresponding to M2 phage and T1 phage, respectively. Metagenomic NGS data was analyzed for pathogens using SURPI+, a clinical version of the SURPI ("sequence-based ultra-rapid pathogen identification") computational pipeline for automated detection of pathogen sequences from mNGS data (*2*). The March 2015 build of NCBI GenBank was used as the reference database for comparison.

Specific threshold cutoffs for organism detection were empirically chosen a priori based on data from prior extensive research-based testing of CSF samples. Briefly, the criteria for pathogen identification and reporting were as follows: (i) for reporting of bacteria, fungi, and parasites, a RPM (reads per million) ratio of ≥10, defined as the RPM_{sample} / RPM_{NTC} for any given taxon (species, genus, or family); (ii) for reporting of viruses, coverage at least 2 non-

contiguous / non-overlapping gene regions of size greater than the read length (140 bp). Viruses corresponding to non-pathogenic flora (e.g., anelloviruses) or contaminants found in the NTC were not reported.

SLEV RT-PCR Confirmation

Confirmation of SLEV detection in the research laboratory and by the California Department of Public Health and U.S. CDC was performed by virus-specific RT-PCR using primers and conditions specified in (*3*). The sequence of the PCR amplicon by Sanger sequencing corresponded to SLEV.

Phylogenetic Analysis

The genomes corresponding to the patient's SLEV, an SLEV strain from a mosquito pool collected in 2016 from Kern County, California, and all 32 complete SLEV sequences available in NCBI GenBank as of December 2016 were aligned using the MAFFT program (*4*) at default settings. Phylogenetic trees were constructed using the neighbor joining algorithm with 10,000 bootstrap replicates in Geneious (*5*).

GenBank Accession Numbers

The genome sequences corresponding to the patient's SLEV and the SLEV from a 2016 mosquito pool collected from Kern County, California have been deposited in NCBI GenBank (accession numbers KY825742-KY825743). Following removal of human sequences, metagenomic reads corresponding to the patient's RNA and DNA libraries and control samples (NTC and PC) have been deposited in the NCBI Sequence Read Archive (SRA) (BioProject PRJNA380606, accession number SRP102510).

References

1. Mongkolrattanothai K, Naccache SN, Bender JM, Samayoa E, Pham E, Yu G, et al. Neurobrucellosis: Unexpected Answer From Metagenomic Next-Generation Sequencing. J Pediatric Infect Dis Soc. 2017 Jan 6:piw066. PubMed http://dx.doi.org/10.1093/jpids/piw066

2. Naccache SN, Federman S, Veeraraghavan N, Zaharia M, Lee D, Samayoa E, et al. A cloud-compatible bioinformatics pipeline for ultrarapid pathogen identification from next-generation sequencing of clinical samples. Genome Res. 2014;24:1180–92. PubMed http://dx.doi.org/10.1101/gr.171934.113

3. Lanciotti RS, Kerst AJ. Nucleic acid sequence-based amplification assays for rapid detection of West Nile and St. Louis encephalitis viruses. J Clin Microbiol. 2001;39:4506–13. PubMed http://dx.doi.org/10.1128/JCM.39.12.4506-4513.2001

4. Katoh K, Standley DM. MAFFT multiple sequence alignment software version 7: improvements in performance and usability. Mol Biol Evol. 2013;30:772–80. PubMed http://dx.doi.org/10.1093/molbev/mst010

5. Kearse M, Moir R, Wilson A, Stones-Havas S, Cheung M, Sturrock S, et al. Geneious Basic: an integrated and extendable desktop software platform for the organization and analysis of sequence data. Bioinformatics. 2012;28:1647–9. PubMed http://dx.doi.org/10.1093/bioinformatics/bts199
